# Supplementary material for: Direct Solar Oven with and without UV Filter vs. Traditional Oven: Effect on Polyphenolic Antioxidants, Vitamins and Carotenoids of Food
Source: Molecules. 2023 Apr 17;28(8):3519. doi: 10.3390/molecules28083519 (PMC10143948; doi:10.3390/molecules28083519)
Supplement: Supplementary file 1 [file molecules-28-03519-s001.zip › molecules-2325206-supplementary.pdf]

Supporting Informations

Table S1. Time and temperature of cooked samples with different baking methods.

sample	time/cooking temperature	electric oven	solar oven	solar oven with filter
carrots	min	25	20	30
	°C	179	178	177
onions	min	23	23	25
	°C	170	175	170
aubergines	min	60	60	60
	°C	137	143	143
pepper	min	30	30	35
	°C	180	180	182
zucchini	min	15	15	12
	°C	175	182	182
chicken	min	25	30	30
	°C	185	183	185
pork loin	min	10	9	10
	°C	176	176	173
cod fish	min	18	18	18
	°C	189	185	177
